# Exploring data science as a tool to advance linked socioecological systems in the context of climate change

**DOI:** 10.1073/pnas.2321149123

**Published:** 2026-10-26

**Authors:** Kathryn Grace, Peter B. Reich, Arun Agrawal, Molly E. Brown

**Affiliations:** ^a^ Department of Statistics and Applied Probability, University of California, Santa Barbara, CA 93106; ^b^ Department of Geography, University of California, Santa Barbara, CA 93106; ^c^ Institute for Global Change Biology and School for Environment and Sustainability, University of Michigan, Ann Arbor, MI 48109; ^d^ Department of Forest Resources, University of Minnesota, St. Paul, MN 55108; ^e^ Keough School of Global Affairs, University of Notre Dame, South Bend, IN 46556; ^f^ Department of Geographical Sciences, University of Maryland, College Park, MD 20742

Theoretical advances, novel analysis methods, and rapidly growing data streams are transforming the understanding of how climate, demographic, and environmental changes affect health and well-being (1). Regardless of levels of development and across sociopolitical contexts, it is critical to gain stronger traction on the role of environmental and global changes on human lives and social systems (2, 3). A growing literature has begun to disentangle the complex web of influences that socioenvironmental relationships constitute.

Focusing on this rich and expanding body of work, the contributions to this special feature use a socioecological systems approach to address at least two major scientific challenges. One, what does available quantitative evidence reveal about how climatic and other global changes affect the vulnerability and responses of different groups of people? And, two, how do ongoing shifts shape attitudes and behaviors (and potentially agency) within and among different social groups from local to global scales? Both questions possess long-standing relevance. But it has become more feasible to address them and expand understanding of human capacity in these systems because available streams of spatiotemporal data at relatively fine resolution offer an unprecedented opportunity to anchor and strengthen knowledge about the effects on ongoing changes through dynamic, interconnected, and multilevel socioecological processes (4, 5). The articles included in this feature all adopt aspects of a socioecological perspective as they each employ, in different ways, frameworks that consider relationships among drivers and responses across multiple scales to explain social patterns and outcomes. Using a socioecological framing is not new and has experienced periods of more or less popularity but has been in a process of expansion as scholars consider adaptation to climate change to include human agency and vulnerability more explicitly into conceptualization and modeling (4, 6). The articles demonstrate the importance and value of using data-science focused approaches to consider multiple scales of socioecological systems to better understand climate impacts and human agency across diverse and vulnerable populations.

Climate change risk and vulnerability^*^ vary based on place but also based on the identities, endowments, and relationships of those who inhabit the place. Because social systems are changing in response to environmental change, and environmental changes are related to shifts in social systems, processes of management and marginalization emerge as part of these interconnected changes. The articles address risk and measurement of risk in different ways but all highlight the importance of considering the unique constraints and opportunities faced by marginalized groups exposed to different climate change impacts (as described by a socioecological perspective). This approach helps demonstrate ways in which the question of human agency as a component of socioecological systems can be explored.

As observed in the articles, applying a socioecological lens requires situating individual behaviors and health or demographic outcomes within multiple levels of interacting social and environmental systems that respond to and impact systems, including weather extremes and climate shifts (e.g., ref. 6). As such, human behaviors and social systems may respond inconsistently compared with a sequencing or ordering anticipated by scientists and policymakers. Specifically, the articles generally address some aspect of human agency as it relates to key topics in demography [e.g., childbearing (8) or mortality (9, 10)] or public health [e.g., child health (11)] and explore how levels of urbanization, education, or development, for example, can be modeled and better understood in the context of local-level experiences climate change. Such a systems-oriented perspective, combined with foundational, empirical scholarship by scientists working on climate, population, and vulnerability, offers important insights for advancing contemporary interdisciplinary population-environment science. In particular, it can help strengthen the integration of data science and more holistic theoretical approaches, both of which are necessary to advance climate change science.

## Purpose of the Special Feature

The aim of this special feature is to examine how data science embedded within holistic social–ecological systems frameworks can advance our understanding of climate change impacts on human populations, health, and justice outcomes. Drawing on diverse data sources across spatial and temporal scales, the contributions demonstrate the potential of these approaches to capture the complex and interconnected dimensions of climate change. The pieces contribute to this goal in different ways—some focus more on methods and measures (e.g., refs. 12 and 13), some on data science (8, 10, 11, 14), and some on repurposing long-standing approaches (9, 15) to consider more spatial or temporal complexity in the human or environmental systems. All incorporate a socioecological perspective that demonstrates the need to consider human-environment processes dynamically, across multiple spatial and temporal scales to better understand the coupled effects of climate and demographic change on health and well-being. Readers will therefore benefit from the application of theory and the development of data science approaches in addition to the unique topical contributions of each article.

Notable in this feature, scholars examine issues of climate change impacts on human population, health and justice outcomes either by telescoping out to holistically examine the global scene (e.g., refs. 10 and 15), or reversing focus to gain insights into specific place-based contexts or issues (8, 11). Telescoping out [Reich et al. (15) is a good example here] gives the “big picture” (and is essential when contemplating climate mitigation) but necessarily cannot address place- and time-specific nuances and context dependencies. of what happens to a specific person in a given location at a given time. In contrast, a highly focused place-based examination [Balk et al. (9) or Brooks et al. (8) are good examples] may not reflect specific statistical associations that are relevant at different spatial scales, and so provide less insight for decision-making and policy planning at more “macro” or global scales, but provides case studies of how justice, vulnerability, and climate change impacts play out on the ground. Additionally, although adaptive management can be planned at coarse scales, whether or not any particular action is in fact adaptive will tend to be local, while to be meaningful, mitigation efforts at local scales need to contribute to global carbon goals [see, for example, Horton et al. (16) work in Science on linking bottom–up and top–down approaches to advance climate change policy]. This Special Feature attempts to engage both “telescoping” and “microscoping” lens—and to meld both approaches together when possible [Mikal et al. (10) and Bassetto et al. (14) explore different aspects of multiple spatial dimensions, for example].

## Contribution of Special Feature as a Collection

The articles included in the special feature consider data at different scales of aggregation, over time and space and hierarchies, to derive measures of climate and weather conditions in ways that align with linked social and ecological systems at different scales. The complexities of quantitatively documenting the way different types of systems interact are described with a focus on the spatial and temporal dimensions of the interactions.

Consistent with efforts to expand population and health transition models to include environmental context, each article considers how environmental conditions constrain and shape social systems responses in their given settings. In terms of broader impacts, the research results highlight policy responses, including different systems-level (as opposed to individual behavioral modifications) opportunities to support the needy and reduce vulnerability to climate change.

For scholars entering into or expanding this interdisciplinary approach to climate change science, the articles provide guide for how to explore different themes—risk management, data aggregation and disaggregation, and actionable findings. They provide models for how to manage data, conduct analyses, and engage with findings in a way that connects disciplines and supports scientific based policy development. Because the topics are diverse and each paper has a unique scientific contribution, they also reveal the complexities and necessity of interdisciplinary climate change research. Each article considers climate change risk and vulnerability at different scales but with attention to data science issues of aggregation and disaggregation (as described below).

Reich et al. (15) provides an overarching perspective on the challenges of climate change mitigation policy as potentially exacerbating social inequalities. As such, the authors suggest and demonstrate with, comparisons of over 40 countries, how micro- and macrosocial and ecological factors must be considered alongside each other to best mitigate the adverse impacts of climate change. The authors thus argue that climate mitigation and social justice are inseparable, because mitigation actions can worsen existing inequalities if not designed with justice in mind and because unchecked climate change (which could, for example, be worsened by avoiding unjust mitigations) will worsen inequalities in different ways and at different scales. It describes how poorer countries and marginalized groups—especially women, children, indigenous peoples, and other disadvantaged communities—face the highest exposure to heat, flooding, drought, food insecurity, and pollution, with severe consequences for health, income, and education. Continued climate warming will drive continued economic productivity losses concentrated in low-income, high-inequality states, tightly linking climate risk to multidimensional well-being. Across energy, transport, buildings, food systems, and nature-based solutions, the paper stresses that mitigation can either reduce or amplify vulnerability and health risks depending on who participates, who pays, and who benefits. Reich et al. provide focused analysis that links climate policy, finance, and carbon pricing to distributive, procedural, and intergenerational justice, arguing that valuing future damages appropriately and targeting benefits to the poor are essential to align mitigation with improved health and development.

Balk et al. (9) provide a case-study, focusing on mortality in New York City. The authors expand standard demographic approaches to investigating mortality to include climate risks and human health vulnerabilities. The authors develop long-term, high-resolution population projections for New York City under the Shared socioeconomic pathways (SSPs) to better understand who will be at risk from future climate hazards, where, and under what social conditions. Using a classic demographic modeling approach—multistate, multiregional demographic model -they project population by age, sex, and race/ethnicity for each borough to 2100 and downscales results to 100 m grids, enabling analysis of neighborhood-level vulnerability and equity. The authors then link changing climate hazards (especially coastal flooding and extreme heat) with projected shifts in population size, location, and composition, emphasizing that vulnerability depends on both exposure and social characteristics. They show that across most SSPs, New York’s future population will be smaller but substantially older, with large increases in people aged 65+, especially women, and with elevated risks of climate hazards, which together heighten risk from heat and disaster events, and raise concerns about isolation and care needs. Thus, their research highlights the compounding effects on humans of climate change and the potential for human vulnerabilities to increase in unforeseen ways depending on local-level climate change outcomes. Considering changing demographic composition in combination with climate risks requires socioecological thinking and the results suggest that multiple dimensions of human impact must be considered for mitigation.

The distributed extendible complementarity (DECO) model is presented in Zaitchik et al. (13) using data from Ethiopia and Mali to understand how climate- and policy-related shocks propagate through networked food systems and affect food security, prices, and human welfare. DECO uses subnational dynamics in food, water, and energy systems, to describe the interactions between droughts, policy shifts, technology adoption, and food aid. In this paper, climate risk is represented mainly through drought-induced crop failures and their cascading impacts on production, prices, and caloric intake across regions. Health and development outcomes are captured through changes in caloric intake, dietary diversity, prices, producer profits, consumer surplus, and regional inequalities. The simulations presented show that different interventions—food aid, cash aid, subsidies, export bans, or transport expansion—can have counterintuitive distributional effects, sometimes benefiting wealthier regions or intermediaries more than food-insecure households. By resolving who gains and who loses under each scenario, the socioecological system framing informs equitable adaptation and development strategies aimed at improving nutrition, livelihoods, and resilience for vulnerable rural and urban populations. This paper provides a unique modeling approach and guide for actually developing complex socioecological systems–based models for analysis and decision-making.

Brown et al. (12) explores the critical role of dataset selection, climate indicators, and baseline periods in translating climate anomalies into actionable knowledge for risk management. Climate extremes directly threaten human development through heatwaves, which cause excess deaths and hinder child development, especially in tropical and urban areas. As climate impacts intensify, decision-makers must navigate complex trade-offs between data resolution and record length to avoid misinforming disaster responses. In this paper, human vulnerability is shown to be highly context-specific, with extreme weather caused by a warming climate already causing significant multisectoral damage. It uses an analysis in France to show systematic vulnerability to extremes heat that led to over 15,000 deaths during a 2003 heatwave, exacerbated by social isolation and a lack of cooling technologies. The findings reveal differences in level of vulnerability and response across social groups.

Firsthand exposure to temperature extremes in Europe, such as the extreme heatwave of 2003 in France described in Brown et al. (8), was shown by Bassetto et al. (14) to influence climate attitudes and electoral support for green policies and parties. The authors find that each additional degree of excess temperature during warm spells increases the probability of worrying about climate change by 4.0 percentage points and voting for green coalitions by 2.8 percentage points. The research highlights significant heterogeneity in vulnerability and response. While highly educated women consistently show the highest proenvironmental attitudes, poorly educated men—who initially exhibit the lowest engagement—show the strongest increases in concern after experiencing heat extremes. Direct experience acts as a powerful symbolic event, bridging gaps for groups previously less engaged due to ideological or lifestyle grounds.

Brooks et al. (8) investigates the impact of humid-heat exposure on fertility in Nigeria, one of the world’s most climate-vulnerable countries. By integrating demographic surveys with high-resolution humid-heat data, researchers examined how heat waves affect conception and pregnancy termination. Hotter conditions initially decrease conception probability by 1.4% but later cause a “rebound” shift in timing. This impact is significantly larger for women in rural areas, those in dryer districts, and those with lower educational attainment. Urban residents show higher resilience to conception shifts, likely due to better access to cooling infrastructure like fans and air conditioning. A 1 deg increase in humid-heat during pregnancy is associated with a 1.73 percentage-point increase in miscarriage because thermal stress disrupts normal nutrition and body temperature regulation, leading to adverse outcomes like preterm birth, low birth weight, and pregnancy loss. These shifts undermine the ability of individuals to conceive under their chosen conditions, potentially leading to social stigma in communities where large families are highly valued. This study illuminates how extremely hot and humid weather may affect human health in coming decades as climate change accelerates.

Mikal et al. (10), in their forward looking article, highlight ways to harness social media data to investigate less explored themes and impacts of climate change on human experiences. They take advantage of the complexity of investigating climate change impacts in combination with the social media data landscape to develop potential new data analysis strategies. Specifically, the authors focus on what types of new climate-health questions or hypothesis can be explored using social media data. They highlight how social media data potentially provides unusually detailed information relevant to the experiences of more marginalized groups in terms of climate change impacts. The proposed qualitative analysis approach to data gathering and interpretation merge multiple disciplines and perspectives while centering the diversity of human experiences. The outcome of the authors efforts is to highlight the potential for social media content to give access to the lived experiences of less studied groups of people quickly and at lower cost than other methods.

Finally, Shukla et al. (11) evaluates the potential of an early warning system to predict birth weights in West Africa using subseasonal to seasonal (S2S) climate data and socioeconomic indicators. Focusing on Burkina Faso, Mali, and Senegal, the authors found that models using only climate predictors—such as precipitation and heat extremes—achieved predictive accuracy comparable to models using detailed, but less available, household survey data. Vulnerability to heat waves is driven by a combination of socioeconomic status and the timing of environmental exposure. Here, the authors use S2S climate variables to identify interannual shocks like droughts and heatwaves that trigger lower birth weights in vulnerable populations. Predictive skill of the model was highest for children with significant in-utero exposure to their mothers’ food insecurity during periods when food stocks are typically depleted. The study demonstrates that high-resolution climate forecasts can provide actionable insights for malnutrition surveillance up to a year in advance. This capability allows decision-makers to implement preventive measures, such as nutritional assistance and counseling, to mitigate the negative impacts of climate anomalies on child development.

## Conclusion

The special feature includes contributions that address how to use and advance social science analysis in combination with geospatial data to develop culturally relevant indicators of climate change mitigation, adaptation, and/or impacts that take advantage of the advances in geospatial data and sciences. The articles provide clear examples of how to develop an interdisciplinary and systems-oriented research that rigorously engages with both social and physical science data and methods. All together these efforts highlight the complexity of human agency in a context of climate change—a perspective that helps to advance social science scholarship as well as scholarship focused on coupled human-environmental systems.

The research in this feature demonstrates how climate change, the greatest social and environmental challenge of our time, demands that science advance data, methods, and theory in innovative ways. In fact, reducing human suffering as a result of climate change requires that scientists expand their tools and data and embrace the need to refine and refute disciplinary assumptions and foundational theories. Scientists can provide vital insight into strategies for mitigating climate change while reducing social injustice—to best accomplish this, they must push the limits of their fields to reveal new scientific possibilities to support new social and policy responses to global warming.

## Footnotes

^*^We note that the language around “vulnerability” varies by discipline. In this special feature and associated research, by vulnerability we are referring to the factors that create or attenuate exposure and subsequent susceptibility to harm in an individual or human system in response to the effects of climate change (7).
